# Comparison of Hypnotic Suggestion and Transcranial Direct-Current Stimulation Effects on Pain Perception and the Descending Pain Modulating System: A Crossover Randomized Clinical Trial

**DOI:** 10.3389/fnins.2019.00662

**Published:** 2019-06-26

**Authors:** Gerardo Beltran Serrano, Laura Pooch Rodrigues, Bruno Schein, Andressa Souza, Iraci L. S. Torres, Luciana da Conceição Antunes, Felipe Fregni, Wolnei Caumo

**Affiliations:** ^1^Post-Graduate Program in Medical Sciences, School of Medicine, Universidade Federal do Rio Grande do Sul, Porto Alegre, Brazil; ^2^Pain and Palliative Care Service, Hospital de Clínicas de Porto Alegre, Porto Alegre, Brazil; ^3^Laboratory of Pain and Neuromodulation, Hospital de Clínicas de Porto Alegre, Porto Alegre, Brazil; ^4^Psychology Department, Universidad Católica de Cuenca, Cuenca, Ecuador; ^5^Department of Nutrition, Health Science Center, Universidade Federal de Santa Catarina (UFSC), Florianópolis, Brazil; ^6^Postgraduate Program in Health and Human Development, La Salle University, Canoas, Brazil; ^7^Berenson-Allen Center for Noninvasive Brain Stimulation, Harvard Medical School, Boston, MA, United States; ^8^Department of Neurology, Beth Israel Deaconess Medical Center, Harvard Medical School, Boston, MA, United States; ^9^Department of Pharmacology, Instituto de Ciências Básicas da Saúde, Universidade Federal do Rio Grande do Sul, Porto Alegre, Brazil

**Keywords:** hypnotic analgesia, transcranial direct-current stimulation, pain threshold, conditioned pain modulation, brain-derivate-neurotrophic-factor, pain

## Abstract

**Objectives:** This paper aims to determine if hypnotic analgesia suggestion and transcranial direct-current stimulation (tDCS) have a differential effect on pain perception. We hypothesized that transcranial direct-current stimulation would be more effective than hypnotic analgesia suggestion at changing the descending pain modulating system, whereas the hypnotic suggestion would have a greater effect in quantitative sensory testing.

**Design:** This is a randomized, double blind and crossover trial.

**Settings:** All stages of this clinical trial were performed at the Laboratory of Pain and Neuromodulation of the Hospital de Clínicas de Porto Alegre.

**Subjects:** Were included 24 healthy females aged from 18 to 45 years old, with a high susceptibility to hypnosis, according to the Waterloo-Stanford Group Scale of Hypnotic Susceptibility, Form C (15).

**Methods:** The subjects received a random and crossover transcranial direct-current stimulation over the dorsolateral prefrontal cortex (2 mA for 20 min) and hypnotic analgesia (20 min).

**Results:** Only hypnotic suggestion produced changes that are statistically significant from pre- to post-intervention in the following outcomes measures: heat pain threshold, heat pain tolerance, cold pressure test, and serum brain-derivate-neurotrophic-factor. The analysis showed a significant main effect for treatment (*F* = 4.32; *P* = 0.04) when we compared the delta-(Δ) of conditioned pain modulation task between the transcranial direct-current stimulation and hypnotic suggestion groups. Also, the change in the brain-derivate-neurotrophic-factor was positively correlated with the conditioned pain modulation task.

**Conclusion:** The results confirm a differential effect between hypnotic suggestion and transcranial direct-current stimulation on the pain measures. They suggest that the impact of the interventions has differential neural mechanisms, since the hypnotic suggestion improved pain perception, whereas the transcranial direct-current stimulation increased inhibition of the descending pain modulating system.

**Clinical Trial Registration:** www.ClinicalTrials.gov, identifier NCT03744897.

**Perspective:** These findings highlight the effect of hypnotic suggestion on contra-regulating mechanisms involved in pain perception, while the transcranial direct-current stimulation increased inhibition of the descending pain modulating system. They could help clinicians comprehend the mechanisms involved in hypnotic analgesia and transcranial direct-current stimulation and thus may contribute to pain and disability management.

## Introduction

The pain and emotion circuits are reciprocally interconnected, providing pro- and anti-nociceptive pain modulation (Price, 2000). Although sensory information is primarily transmitted by the ascending pathway, providing the sensory components of the pain experience, top-down neuromodulator techniques can affect both ascending and descending pain processing pathways. These approaches include non-invasive brain stimulation (NIBS) methods, which can alter aberrant activities within the pain processing circuit (e.g., transcranial direct-current stimulation [tDCS]), and psychological pain interventions, which improve the cognitive and emotional components of pain (e.g., meditation and hypnotic suggestions).

Previous studies have shown the efficacy of tDCS for the treatment of various chronic pain conditions (i.e., fibromyalgia, phantom pain, and trigeminal neuralgia) (Fregni et al., 2005; Nitsche et al., 2008). The primary target to apply the tDCS has been the primary motor cortex (M1) (Hou et al., 2016). The stimulation of M1 enhances the strength of the descending pain modulating system (DPMS) in both, healthy subjects (Viana et al., 2014; Silva et al., 2017) and patients with chronic pain (Rainville, 1997; Hou et al., 2016). In addition, the dorsolateral prefrontal cortex (DLPFC) is a target for improving pain sensations and the emotional aspects linked to pain (Fregni et al., 2005; Nitsche et al., 2008; Silva et al., 2017). According to recent studies of fibromyalgia, anodal tDCS over the left DLPFC reduced pain sensations and fatigue (Silva et al., 2017), improved cognitive performance (Santos et al., 2018), and the performance of tasks related to attentional networks (Silva et al., 2017). In addition, prefrontal tDCS might alter the function of emotion-related information processing circuits (Zhao et al., 2017). Specifically, anodal tDCS applied to the left DLPFC, with the cathode over the right DLPFC has been shown to either enhance neural activity and/or reduce neural activity in the right DLPFC (Brunoni et al., 2012).

The DLPFC is also involved in pain modulation through multiple psychological processes. As such, it is activated in experimental pain studies. For example, individuals that received instructions to suppress pain increased activation of bilateral, particularly in the left – DLPFC (Santarcangelo, 2014). Besides, the bilateral tDCS, with anodal stimulation over DLPFC increased the connectivity strength in both regions, thalamus and right anterior insula (Müller et al., 2012). While a single session of tDCS over the left DLPFC significantly increased the heat pain threshold in fibromyalgia (Silva et al., 2017). Further to the factors related to the target to apply the stimulation, other factors can influence the tDCS effect, such as the type and duration of stimulation (anodal or cathodal), schedule and number of repetitive stimulations. In addition, according to previous studies, its effect is likely state-dependent neuroplasticity, since the brain-derived-neurotrophic-factor (BDNF) (Sandrini et al., 2000; Santos et al., 2018) predicted the impact of tDCS on short-term memory in patients with fibromyalgia (Santos et al., 2018) and the disability due to pain after hallux valgus surgery (Yarnitsky et al., 2010).

Regarding to hypnotic analgesia, the literature reports different responses to pain perception in highly susceptible subjects. Its effects include a decreased H-reflex amplitude (Santarcangelo, 2014) and decreased subjective pain perception. Also, it reduces the neuronal activity of the primary somatosensory cortex (SI) (Nitsche et al., 2008). According to a meta-analysis, which included both laboratory and clinical studies, the effect size (ES) of hypnotic analgesia was moderate (ES = 0.71) (Montgomery et al., 2000). The hypnotic suggestions can increase activity on anterior cingulate cortex, which is identified as an “important part of the executive network, as it is involved in selective attention, learning and conflict resolution” (Müller et al., 2012). Its effect on the thalamus is also associated with an “activation of a critical node in the motor path from basal ganglia to higher motor areas” (Müller et al., 2012). In addition, studies have shown that during the cold-pressor test (CPT), hypnotic analgesia increased pain tolerance by a mean of approximately 62% and reduced both pain perception and the nociceptive reflex to 65% of the baseline reflex area (Sandrini et al., 2000). However, diffuse noxious inhibitory control (DNIC) provoked by the CPT (Sandrini et al., 2000) was less effective during hypnosis than without hypnosis. A study suggested that the inhibition of DNIC does not involve spinal motoneuron excitability (Yarnitsky et al., 2010). In short, these results, together with those of earlier studies (Willer et al., 1984; Roby-Brami et al., 1987), suggest that the inhibition of DNIC also involves supraspinal structures. During the last decade, the human DNIC counterpart has been identified and is referred to as conditioned pain modulation (CPM) (Le Bars et al., 1979).

As the application of tDCS can modulate thalamocortical synapses in a top-down manner, and the hypnotic suggestion can improve pain perception new insights are needed to compare how each one these two techniques changes the pain perception and the descending pain-modulating function. Thus, this study investigated whether the tDCS would be more effective than a hypnotic suggestion for improving the inhibitory functions of the DPMS, as assessed by reported changes on the Numerical Pain Scale (NPS ranging from 0 to 10) during the CPM test. And so, this study tested the following hypotheses: (i) hypnotic suggestion would have a superior effect on pain perception in response to the Quantitative Sensory Testing (QST) compared with anodal tDCS applied to the left DLPFC and cathodic tDCS applied to the right DLPFC, as assessed by changes in the heat pain threshold (Δ-HPT), heat pain tolerance (Δ-HPTo) and the cold pressure test (Δ-CPT). (ii) Anodic tDCS applied to the left DLPFC and cathodic tDCS applied to the right DLPFC would be superior to hypnotic suggestion at altering the DPMS function, as assessed by the delta (Δ)-value of the change on the NPS (scale of 0–10) during a CPM-test. In addition, we evaluated the influence of Δ-BDNF on the effects of tDCS and hypnotic suggestion and performed exploratory analyses of the relationships between state anxiety and the Δ-value of the NPS (0–10) during the CPT and between Δ-BDNF and the Δ-value of the NPS (0–10) during the CPM test, according to treatment mode.

## Materials and Methods

### Design Overview, Setting, and Participants

**FIGURE 1:**
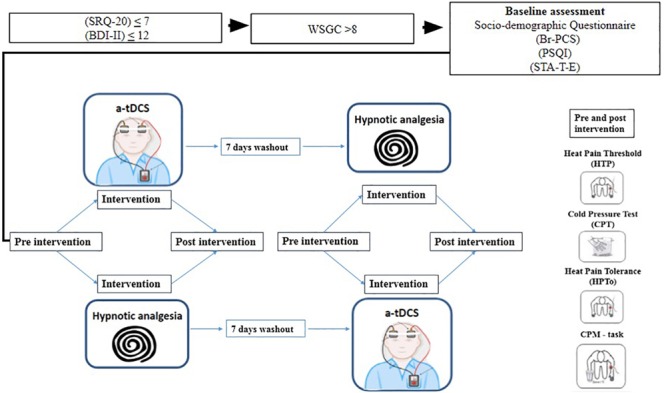
Timeline of procedure of study. Self-Reporting Questionnaire (SRQ-20); Beck Depression Inventory (BDI-II);Waterloo-Stanford Group Scale of Hypnotic Susceptibility (WSGC); Brazilian Portuguese Pain Catastrophizing Scale (Br-PCS); Pittsburg Sleep Quality Index (PSQI); State-Trait Anxiety Inventory (STA-T-E).

All subjects provided written informed consent for their participation in this randomized double blind crossover clinical trial, with a 1:1 allocation ratio. The protocol was approved by the Institutional Review Board (IRB nos. 63863816000005327 and 16-0635) and conducted according to the Declaration of Helsinki. Recruitment was undertaken in the time from July 2017 to November 2018. To assess clinical and psychological characteristics, we used a standardized questionnaire and administered scales validated to the Brazilian population. Additionally, we collected behavioral measurements (i.e., pain assessments). De-identified data relating to intervention and primary outcomes will be made available on request to WC (wcaumo@hcpa.edu.br) with no time restriction. The timeline of study is presented in Figure 1.

### Subjects

The volunteers were recruited from the general population by advertisements posted in the universities, on the internet and in public places in the city of Porto Alegre, Brazil. Subjects were considered eligible to participate if they were healthy women with more than 11 years of education, ranging between 18 to 45 years old. Individuals were excluded if they presented hearing impairment or formal contraindication to transcranial direct-current stimulation (tDCS), according to current guidelines.

During the first contact, the researcher performed the Waterloo-Stanford Group Scale of Hypnotic Susceptibility Form C (Bowers, 1998) and the subjects completed a structured questionnaire that assessed the following variables: current acute or chronic pain conditions, use of analgesics in the past week, rheumatologic disease, clinically significant or unstable medical or psychiatric disorder, history of alcohol or substance abuse in the past 6 months, neuropsychiatric comorbidity, and use of psychotropic drugs. They were excluded if answered any of these questions positively. Subjects with a score greater than or equal to 8/12 on the Waterloo-Stanford Group C Hypnotic Susceptibility Scale (WSGC) were included in the later phases of the investigation, while subjects with Beck Depression Inventory (Warmenhoven et al., 2012) scores higher than 12 were excluded (Li et al., 1975), as were those with positive screening higher seven for minor psychiatric disorders (somatic symptoms, depressive moods, depressive thoughts, and decreased energy) on the World Health Organization (WHO) Self-Reporting Questionnaire (SRQ-20).

### Experimental Protocol

This was a double blind randomized crossover trial. On admission to the study, participants were randomized to initially receive either tDCS or hypnotic analgesia sessions. For the tDCS condition, the anode electrode was positioned over the left dorsolateral prefrontal cortex (DLPFC) and a cathode electrode on the right (DLPFC). A constant current of 2 mA was applied for 20 min, with initial and ending ramps of 30 s long stimulation. The hypnotic analgesia session consisted of a 10 min long standard induction. The protocol began with a set of suggestions to subjects to focus their attention on a single stimulus and they were encouraged to control their breathing, guiding subjects to progressive relaxation. After that, suggestions were given for comfort, in which the patient had to imagine being in a quiet and peaceful place. On the 10 final minutes of the induction, the hypnotic suggestions for analgesia were given targeting the decrease of the subject’s pain and controls over her own sensations. As suggestion for hypnotic analgesia the patient was told that he no longer would feel pain. His mind would be able to control the sensations of his own body, preventing pain. This suggestion was based on Jensen’s approach for hypnotic analgesia (Jensen, 2011). After the first intervention, to stimulate pain and determine the CPM, we used the difference between the pain score on NPS (0–10) QST during cold water immersion (QST+CPM) and the temperature of the point at which subjects felt 6/10 pain on the NPS scale (during the initial time period). To determine heat thermal thresholds (HTT), heat pain threshold (HPTh), and heat pain tolerance (HPTo) during the CPM task the QST was performed. The participants remained seated, and a thermode was positioned on the forearm of the dominant side of the body. The temperature started at 30°C, and the thermode was heated at a rate of 1.0°C/s to a maximum of 51°C, when the temperature began to drop. Besides that, the CPT was conducted to determine on subject’s’ response due to the physical cold stimulus *per se* and the reaction due to the cold pain. During the test, the participant was asked to immerse the dominant hand in ice-saturated water for a maximum of 2 min. The participant returned for a second experimental session to receive the alternative intervention. The order of the experimental sessions was counterbalanced and separated by at least 7 days to avoid carryover effects of the initial stimulation protocol.

### Randomization

Randomized numbers in a 1:1 ratio were generated to allocate each participant to either the HSA or tDCS group. The randomization table was generated by appropriate software. Envelopes were prepared for randomization process and sealed with the subject’s #24 sequence number on the outside of the envelope. The allocation was concealed so no investigator was aware of treatment allocations and therefore had control over the randomized order of patients.

### Blinding

To control possible biases, the following strategies were established. Participants were instructed on all aspects related to the interventions during the evaluations. Two independent evaluators who were not aware of the treatment were trained to do the assessments. The brown envelopes were prepared before starting the study, sealed, initialed and numbered sequentially. The envelope contained the allocation interventions and was opened only after the participant had given her informed consent to participate in the study. The subject’s name and number were immediately sent to those responsible for controlling the randomization process. The blinding was gauged at the end of each evaluation.

### Interventions

#### Transcranial Direct-Current Stimulation

Transcranial direct-current stimulation was applied using Brain Monitoring and Stimulation Technologies (NE, Neuroelectrics Barcelona Sl, model Starstim). Cathodal and anodal electrodes covering an area of 25 cm^2^ each were surrounded by a water-soaked sponge. Electrodes were placed at spatial positions F3-(Anodal) DLPFC – L and F4-(Cathodal) DLPFC – R, according to the international 10–20 system for electroencephalogram electrode placement (Derbyshire et al., 2004) that are commonly considered surface locations above the mid-dorsolateral prefrontal cortex (Homan et al., 1987). Stimulation was delivered at an intensity of 2 mA for 20 min, including a 30 s ramp-up to 2 mA at the start and a 30 s ramp-down to 0 mA at the end. During stimulation, participants were asked to relax while the upper limb was supported in a comfortable position.

#### Hypnotic Analgesia Suggestion Protocol

The techniques of hypnosis developed for this study were based on the classical approach developed by the American clinician and Ph.D. Mark P. Jensen. The hypnotic induction protocol was standardized to be equally applied to all subjects. The standard hypnotic protocol begins with an induction that is associated with breathing and relaxation, where subjects receive suggestions to focus their attention on a single stimulus. Then, direct suggestions are given for comfort and pain management (Patterson and Jensen, 2003). The duration of experimental manipulation (induction + suggestions) is 20 min.

The protocol of suggestion followed these standardized steps, read by the researcher:

“*And from now on, you will no longer feel any more pain... you will not feel any kind of pain after waking up. Your mind will be able to control the sensations of your whole body... Your mind controls the sensations of your body... after you wake up, you will no longer feel any kind of pain... From now on... and after you wake up you will no longer feel any kind of pain... I will count from one to ten and you will feel no pain anymore... your mind will control all the sensations of your body and after waking up you will not feel pain*.”

1: “...*you’re feeling even more relaxed and comfortable...*”
2: “...*you will get even more relaxed and feeling good...*”
3: “...*feeling better and better, you will not feel pain after you’re awake...*”
4: “...*even more relaxed...after waking up, a heat stimulus will be placed on your forearm...and your hand in the ice...and you will not feel any kind of pain...*”
5: “...*will be relaxed and painless...*”
6: “...*your mind will control all the sensations of your body...will control it and you will no longer feel pain after you wake...*”
7: “...*you relax even more...feeling very well...you relax and have good sensations...*”
8: “...*very deep, you will not be able to feel any kind of pain in the periphery of your body when you are awake...*”
9: “...*you will not feel pain...your brain controls all the sensations of the periphery of your body...you will not feel any kind of pain...*”
10: “...*your brain now controls all your sensations of your body...and you will not feel pain after you wake up...feel even more relaxed and comfortable...when you wake up you will no longer feel any kind of pain.*”

“*Now you are very relaxed and feeling comfortable, but very soon you will wake up. I will count from one to ten and to each number you will wake up even more...I will count from one to ten, and you will wake up, feeling good...and will no longer feel any kind of pain...I will count from one to ten and you will wake up, and you will not be able to feel any kind of pain in your body...one...you are gradually coming back and feeling your own body...two...you are gradually coming back...and you can remember that you will no longer feel any kind of pain...three...you can slowly feel your body and the energy increasing...four...you are waking up and feeling your body on the chair...five...halfway through...after waking up you will no longer feel any kind of pain...six...feeling good and slowly waking up...seven...slowly feeling the environment and the movements of your body...eight...you are almost awake...when I get to ten you will wake up feeling good and your brain will not allow you to feel any kind of pain...nine...you are waking up and being aware of your surroundings...waking up more and more...ten...now you wake up feeling good...open your eyes*.”

### Instruments and Assessments

The tools used to evaluate psychological state and hypnotic suggestion were validated to the Brazilian population. Two psychologists were trained to perform the psychological tests and scale of susceptibility. The Waterloo-Stanford Group Scale of Hypnotic Susceptibility, Form C (Carvalho et al., 2008) was used to assess hypnotic susceptibility, and depressive symptoms of patients were assessed by the depression inventory from Beck (BDI-II) (Warmenhoven et al., 2012). The Pain Catastrophizing Scale (PCS) measures a patient’s catastrophizing, defined as “an exaggerated negative “mental set” brought to bear during actual or anticipated pain experience” (Sullivan et al., 2001). Central Sensitization Inventory (BP-CSI) is an instrument used to identify patients with central sensitization syndrome (CSS) and central sensitization (CS) symptoms. The self-report questionnaire (SRQ-20) was used to measure minor psychiatric disorders, somatic symptoms, depressive mood, depressive thoughts, and decreased energy, and the Pittsburgh quality of sleep index to assess sleep quality, as higher scores indicate worse sleep quality (Buysse et al., 1989). We used the refined version of the State-Trait Anxiety Inventory (STAI) (Kaipper et al., 2010) using the Rasch model, which derived STAI-Form X scales from shorter state traits without threshold disorders and for differential element performance (DIF) problems. Scores on the state and trait evaluations vary from 13 to 52 and from 12 to 36, respectively. A standardized questionnaire was used to evaluate demographic data and medical comorbidities.

### Outcomes

The primary outcomes were the NPS (0–10) during the conditioned modulated pain (CPM task) as assessed by the Δ-CPM (i.e., post-minus pre-intervention) and change on NPS (0–10) during the cold pressure test (Δ-CPT). The secondary outcomes were Δ-HPT and Δ-HPTo assessed by quantitative sensory testing (QST).

### Outcomes Assessment

In this study, we evaluated pain as a response to a nociceptive stimulus using quantitative sensory testing (QST), including the conditioned pain modulation task (CPM task) and the cold pressure test (CPT).

(a) To perform QST, we have used a computerized version of the thermostat (Heat Pain Stimulator 1.1.10, Brazil) (Schestatsky et al., 2011) to determine heat thermal thresholds (HTT), heat pain threshold (HPTh), and heat pain tolerance (HPTo) during the CPM task. The participants remained seated, and a thermode (30 mm × 30 mm) was positioned on the forearm of the dominant side of the body. The temperature started at 30°C, and the thermode was heated at a rate of 1.0°C/s to a maximum of 51°C, when the temperature began to drop. For the HTT, the participants were asked to press a button when they “felt the first heat sensation” and were asked to press a button when they “felt the first heat pain.” The heat thermal threshold and heat pain threshold were determined by the average of three evaluations with a 40 s interval between them. Also, we assessed the HPTo.
(b) To measure the CPM test, we evaluated the pain intensity in two tonics HPT test stimuli separated by a CPM test. We used the HPT as conditioning pain stimulus to elicit a prolonged pain sensation to trigger CPM. The CPM test consisted of immersion of the non-dominant hand in cold water at a temperature of 0–1°C for 1 min. A thermostat was used to control the temperature variation and to maintain the water temperature. The QST procedure was introduced after 30 s of cold-water immersion. To determine the CPM, we used the difference between the pain score on NPS (0–10) QST during cold water immersion (QST+CPM) and the temperature of the point at which subjects felt 6/10 pain on the NPS scale (during the initial time period).
(c) CPT was conducted to determine on subjects’ response due to the physical cold stimulus *per se* and the reaction due to the cold pain (Mitchell et al., 2004). During the test, the participant was asked to immerse the dominant hand in ice-saturated water for a maximum of 2 min (Petersen-Felix et al., 1994; Koltzenburg et al., 2006). The temperature of the ice water was measured, and across all tests, it ranged from 9 to 10°C. Perceived pain intensity was rated continuously on a 0–10 electronic visual analog scale (VAS) with the non-dominant hand and stored electronically for 2 min for subsequent analysis of peak pain intensity. If pain was intolerable before 2 min, the subject could withdraw, in which case pain intensity was considered maximal until the end of the 2 min period (Mitchell et al., 2004).
(d) To test serum levels of BDNF, blood samples were collected at baseline at pre-intervention and post-intervention. Using a ChemiKine BDNF Sandwich ELISA kit, CYT306 (Chemicon/Millipore, Billerica, MA, United States), serum BDNF was determined by the Enzyme-linked Immunoabsorbent Assay (ELISA). The lower detection limit of the kit is 7.8 pg/ml for BDNF.

### Sample Size

The sample size was estimated using the G^∗^Power software, based on a previous study with a similar methodology (Effect of hypnotic suggestion on fibromyalgia pain: Comparison between HSA and relaxation, Antoni Castel). The calculus indicated that a sample size of 12 individuals would be necessary to detect a 3-point difference in the numerical scale of pain (average SD 0.59) (NPS) in pain levels to nociceptive stimuli, with a power of 0.95 and an α of 0.05. To ensure the power of the study, 15% was added in case of possible losses, totaling 24 subjects (12 per group). This also provided a power to detect a meaningful effect size [determination coefficient (*f*^2^) = 0.2] to detect differences between the two groups in the other outcomes.

The randomization table will be generated by computer program (Randomlogue). Random codes were placed in brown envelopes sealed. The sequence number is shown on the outside of the envelope.

### Statistical Analysis

Descriptive statistics were used to summarize the main socio-demographic features of the sample. *T*-tests for independent samples were used to compare continuously between groups. To compare the change within a group, Wilcoxon Signed Ranks tests were used. To test for normality, we used the Shapiro–Wilk test. After verifying the corresponding assumptions, a mixed ANCOVA model was used to analyze the main effect of interventions. Factors were the intervention (tDCS and hypnotic suggestion) and the order of the treatments. The order of interventions was included in the model to assess a possible carryover effect produced by the two sequences of treatment to which all subjects were randomly assigned. The outcomes were evaluated using the mean variation for delta (Δ)-values, post-intervention minus pre-intervention) of the following measures: score on the NPS (0–10) during the CPT, change on NPS (0–10) during the CPM task, HPT, and HPTo. The covariate included in all models was the change on serum BDNF (Δ-value, post-intervention minus pre-intervention). We performed all analyses by two-tailed tests, and they were corrected for multiple comparisons using the Bonferroni test. Within groups, the standardized mean difference (SMD) was computed in terms of the ratio between the mean change and the pool of baseline standard deviation (SD). The SMD was interpreted as follows: small, 0.20–0.4; moderate, 0.50–0.70; and large, 0.80 or higher, with respective confidence interval (CI). We accepted a type I error of 5%. To perform the analyses, we used the software SPSS version 22.0 (SPSS, Chicago, IL, United States).

## Results

### Demographic and Characteristics of the Subjects

**FIGURE 2:**
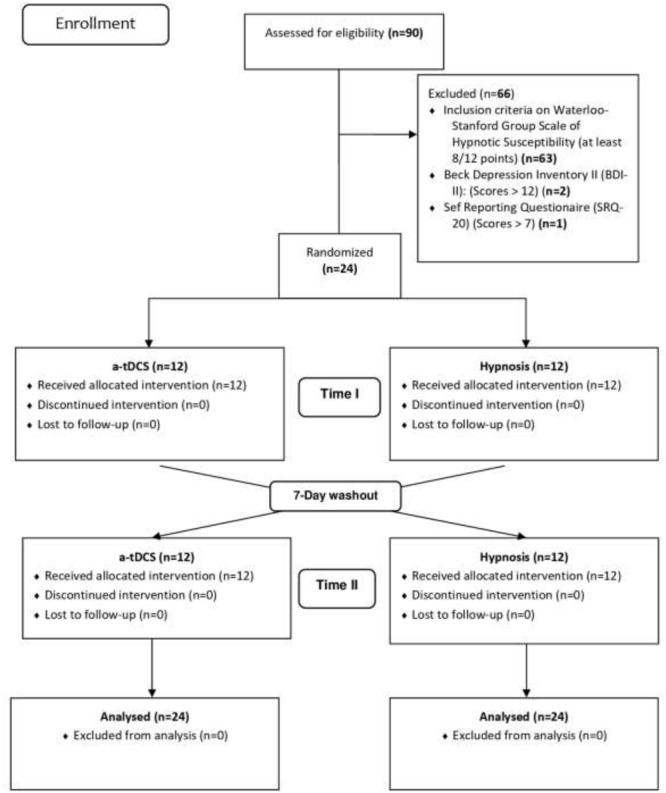
Flowchart of study.

A total of 90 subjects were recruited to participate in this study. After applying the Waterloo-Stanford Group C (WSGC) Scale of Hypnotic Susceptibility, using a cutoff point (8/12) for susceptibility to hypnosis, 27 subjects were selected for the hypnosis experiment. These 27 subjects underwent screening for the presence of minor psychiatric disorders, as determined by the Self-Reporting Questionnaire (SRQ-20) and the Beck Depression Inventory-II (BDI-II). Three subjects were excluded because we identified the presence of minor psychiatric disorders or scores on the BDI-II that were higher than the cutoff point of 12. The final sample included 24 subjects who were randomized to receive either tDCS or hypnotic suggestion for analgesia. For each group, 12 participants were randomized and assigned in a crossover manner to participate in the two sequences of treatment. For all outcomes, 24 subjects were analyzed by the arm, as shown in the study flowchart (see Figure 2).

### Primary and Secondary Outcomes: Univariate Analyses

The socio-demographic characteristics of the subjects according to the sequence allocation were comparable and are shown in Table 1. Twelve subjects were allocated to trial I, which received tDCS first, and twelve subjects were allocated to trial II, which received hypnotic suggestions for analgesia first. All subjects completed the protocol to which they had been randomized.

**Table 1:** Demographic and clinical characteristic of the sample.

	tDCS (*n* = 12)	Hypnotic suggestion (*n* = 12)	*P*-value^∗^
**Demographic**			
Age (years)	26.00 (7.66)	27.00 (10.66)	0.85
Level of education (years)	14.09 (2.76)	14.92 (2.76)	0.47
**Psychological and sleep quality measures**			
Waterloo-Stanford Group Scale of Hypnotic Form C (WSGC)	8.73 (1.00)	8.61 (1.04)	1.00
Self-Reporting Questionnaire (SRQ-20)	3.27 (1.34)	3.15 (2.30)	0.88
Beck Depression Inventory – BDI – II	6.08 (4.68)	4.95 (3.35)	0.34
Pain Catastrophizing Scale – PCS	10.66 (9.93)	10.91 (11.29)	0.93
Central Sensitization Inventory – BP – CSI	23.04 (10.34)	23.54 (9.74)	0.86
State-Trait Anxiety Inventory – STAI			
State-Anxiety (STAI)	21.54 (6.82)	22.37 (5.60)	0.64
Trait-Anxiety (STAI)	18.91 (3.62)	19.75 (4.54)	0.48
Pittsburgh Sleep Quality Index – PSQI	4.75 (1.79)	5.33 (2.18)	0.32

*Data are presented as mean and standard deviation (SD) according to group in trial I (*n* = 24). State-Trait Anxiety Inventory (STAI). ^∗^Compared using *t*-test for independent samples.*

The within and between groups comparisons of psychophysical measures (HPT, HPTo, CPT, and Δ-value of NPS during the CPM task) and serum levels of BDNF, according to the intervention, are presented in Table 2. Comparisons revealed that only hypnotic suggestions for analgesia produced significant changes between the pre and post-intervention measures for HPT, HPTo, CPT, and serum BDNF levels.

**Table 2:** Psychophysical tests (HPT, HPTO, CPT, CPM-task, and BDNF) according to intervention group.

	Mean (SD) before intervention	Mean (SD) after intervention	Δ-value	*P*-value £: between group	*P*-value ¥: within group	Effect size
**Heat pain threshold (HPT) °C**						
tDCS (*n* = 12)	38.50 (2.12)	39.69 (1.81)	1.10 (1.62)	0.11	0.05	0.51
Hypnotic suggestion (*n* = 12)	38.41 (1.47)	41.11 (3.47)	2.67 (1.62)		0.00	1.81
**Heat pain tolerance (HPTO) °C**						
tDCS (*n* = 12)	44.74 (2.44)	44.98 (1.78)	0.23 (1.76)	0.00	0.70	0.09
Hypnotic suggestion (*n* = 12)	44.32 (1.96)	46.07 (2.84)	1.74 (2.22)		0.00	0.89
**Score on NPS (0-10) during the (CPT)**						
tDCS (*n* = 12)	6.850 (2.26)	6.34 (2.67)	–0.50 (1.19)	0.00	0.47	0.22
Hypnotic suggestion (*n* = 12)	7.367 (2.06)	5.26 (2.78)	–2.10 (1.80)		0.00	1.01
**Change on NPS (0-10) during the (CPM-test)**						
tDCS (*n* = 12)	–0.79 (2.84)	–1.08 (2.70)	–0.29 (1.75)	0.83	0.73	0.10
Hypnotic suggestion (*n* = 12)	–1.83 (1.85)	–1.42 (2.74)	0.42 (1.30)		0.76	0.22
**Brain-derived neurotrophic factor (BDNF)**						
tDCS (*n* = 12)	39.81 (19.17)	58.96 (35.82)	13.20 (35.72)	0.00	0.25	0.68
Hypnotic suggestion (*n* = 12)	40.18 (22.04)	29.68 (16.63)	–14.49 (34.63)		0.01	0.66

*Data are presented as mean and standard deviation (SD) and delta [Δ-value of means (post-intervention minus pre-intervention)] (*n* = 24). Celsius degree (°C); Numerical Pain Scale (NPS 0-10),£comparison between group by Wilcoxon-Mann–Whitney. ¥ Comparison within group by Wilcoxon Signed Ranks tests. The effect size within group as assessed by the standardized mean difference (SMD) was computed in terms of the ratio between the mean change and the baseline standard deviation (SD).*

### Primary Outcomes: Multivariate Analyses

#### Intervention Effect During the CPT

A mixed analysis of covariance (ANCOVA) model revealed a significant main effect of the intervention on the Δ-value of NPS during the CPT (*F* = 15.98; *P* < 0.00). An order effect was not observed (*F* = 0.38; *P* = 0.54). The covariate included in the model was the change in serum BDNF level (Δ-value, post-intervention minus pre-intervention). The results of this analysis are presented in Table 3. In the tDCS group, the mean (SD) of the Δ-value of NPS during the CPT (mean post-intervention minus pre-intervention) without the adjustment for the Δ-BDNF value was -0.50 (1.19), while the mean (SD) with adjustment for the Δ-BDNF value was -0.29 (1.23). The pain perception due to the physical cold stimulus increased within the tDCS group by 42%. The effect size of this increment was 0.22 [mean difference: 0.50/1.19 (SD)]. In contrast, in the hypnotic suggestion group, the mean (SD) of the Δ-value of NPS during the CPT without adjustment for the Δ-BDNF value was -2.10 (1.80), while the mean (SD) with adjustment for the Δ-BDNF value was -2.29 (1.74). Pain perception decreased within the hypnotic suggestion group by 8.30%. The effect size of this decrement was 0.10 [mean difference: 0.19/1.80 (SD)].

**Table 3:** Mixed ANCOVA model to assess the treatment effect between groups on Δ-value of the primary outcomes measures [Δ values of the NPS (0–10) during CPT and the change on NPS (0–10) during the CPM-test] (*n* = 24).

	β	SEM	*df*	*t*	*P*-value	CI 95%
**Dependent variable: Δ-score on NPS (0–10) during cold pressure test**			
Intercept	–2.41	0.415	32.06	–5.81	0.00	(-3.26 to -1.57)
Order of intervention	0.28	0.464	43.79	0.61	0.54	(-0.65 to 1.22)
Intervention tDCS	1.92	0.480	42.70	3.99	**0.00**	(0.95 to 2.89)
Hypnotic suggestion 0^reference^							
Δ-BDNF (post intervention minus pre-intervention)	–0.009	0.006	40.84	–1.36	0.17	(-0.02 to 0.004)
**Dependent variable : Δ-change on NPS (0–10) during CPM-test**			
Intercept	1.14	0.523	43.086	2.18	0.03	(0.09 to 2.19)
Order of intervention	–0.11	0.736	41.06	–0.15	0.88	(-1.60 to 1.37)
Intervention tDCS	–1.50	0.715	40.01	–2.09	**0.04**	(-2.94 to -0.05)
Hypnotic suggestion 0^reference^							
Δ-BDNF (post intervention minus pre-intervention)	0.04	0.0126	24.33	3.20	**0.00**	(0.014 to 0.07)
***Interaction between Δ-BDNF vs. intervention***						
Δ-BDNF × tDCS	–0.03	0.0192	41.92	–1.61	0.11	(-0.07 to 0.007)
Δ-BDNF × hypnotic suggestion 0^reference^						

*Brain derived neurotrophic factor (BDNF); β [beta value shows the estimated effect of the intervention (factor) and covariates]; Standard error for mean (SEM); degrees of freedom (df).*

**FIGURE 3:**
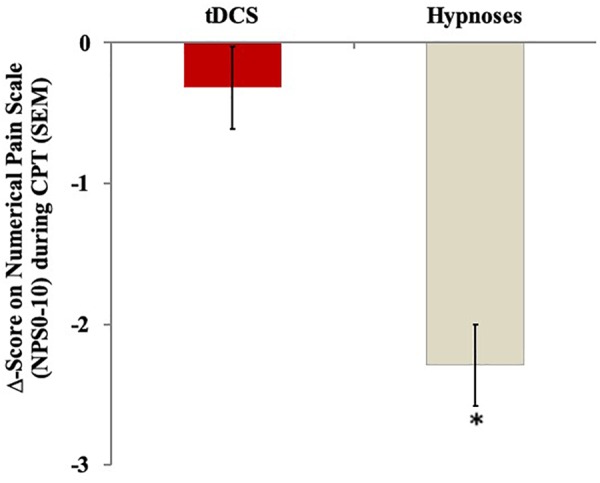
The change in Numerical Pain Scale (NPS 0–10) during cold pressure test with water at 0–10°C, assessed by the Δ-value (score post intervention minus pre-intervention) in the two experimental groups. The error bars indicate standard error of the mean. Asterisk indicates difference between two intervention groups. All comparisons were performed by a mixed analysis of variance model, followed by the Bonferroni test for *post hoc* multiple comparisons.

The mean in the NPS (0–10) during the CPT is presented in Figure 3. A mixed ANCOVA model revealed a significant main effect of interventions on the Δ-value of NPS (0–10) during the CPT (*F* = 15.98; *P* < 0.00). An order effect was not observed (*F* = 0.38; *P* = 0.54), neither influences in the change of serum BDNF.

**FIGURE 4:**
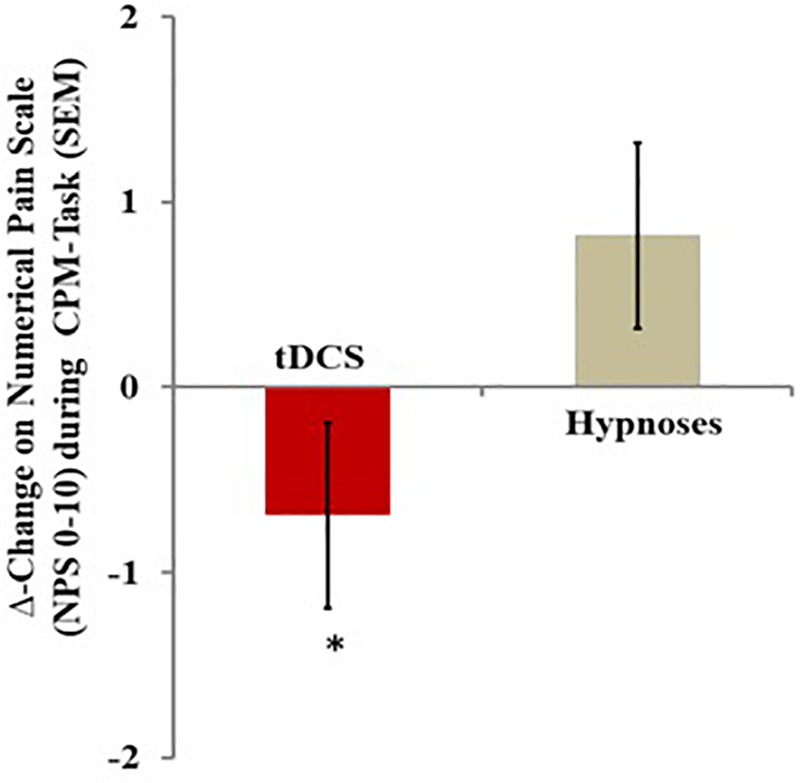
The change in NPS (0–10) during CPM-test, assessed by the Δ-value (score post intervention minus pre-intervention) in the two experimental groups. The error bars indicate standard error of the mean. Asterisk indicates difference between two intervention groups. All comparisons were performed by a mixed analysis of variance model, followed by the Bonferroni test for *post hoc* multiple comparisons. Numerical Pain Scale (NPS 0–10).

#### Intervention Effect on the CPM Test

A mixed ANCOVA model revealed a significant main effect for treatment (*F* = 4.32; *P* = 0.04) when we compared the Δ-values of NPS during the CPM test (mean post-intervention minus mean pre-intervention) between the tDCS and hypnotic suggestion groups. The result of the analysis adjusting for the influences of Δ-BDNF revealed that changes in this neurotrophic factor increased the inhibitory function of the DPMS in the tDCS group, whereas an opposing effect was observed in the hypnotic suggestion analgesia group. In the tDCS group, the mean (SD) of the Δ-value of NPS during the CPM test without adjusting for the Δ-BDNF value was -0.29 (1.75), while the mean (SD) with adjustment for the Δ-BDNF value was -0.45 (1.56). In the tDCS group, BDNF increased the inhibitory function of the DPMS by 39.20%. The effect size of this increment on inhibitory function within the group, as assessed by SDM, was 0.42 [mean difference: 0.74/1.75 (SD)]. In contrast, in the hypnotic suggestion group, the mean (SD) of the Δ-value of NPS during the CPM test without adjustment for the Δ-BDNF value was 0.42 (1.30), while the mean (SD) with adjustment for the Δ-BDNF value was 1.11 (3.34). In the hypnotic suggestion group, BDNF decreased the inhibitory function of the DPMS by 165%. The effect size of this decrement on inhibitory function within the group, as assessed by SDM, was 0.53 [mean difference 0.69/1.30 (SD)]. When interpreting these results, it is important to recognize that a higher Δ-value of NPS during the CPM task indicates the reduced inhibitory function of the DPMS. These findings show that the effect of BDNF on the DPMS is likely to be related to the effects observed for the interventions (tDCS or hypnotic suggestion) on the Δ-BDNF, as tDCS increased the BDNF level while hypnotic suggestion reduced the BDNF level (Table 2). When we analyzed the interaction between the Δ-BDNF value and the intervention group, we did not find an interaction between Δ-BDNF and intervention (*F* = 0.07; *P* = 0.78) (Table 3). Based on these results, the changes in the neuroplasticity mechanisms induced by the type of intervention can explain their effects on the inhibitory function of the DPMS, which increased with tDCS and decreased with hypnotic suggestion.

### Intervention Effect on the Secondary Outcome: HPT and HPTo

The mixed ANCOVA analyses of the main effects of the intervention on the Δ-value of the HPT and HPTo are presented in Table 4. The mixed ANCOVA revealed a main effect of group on the Δ-HPTo (*F* = 5.10; *P* = 0.02). Hypnotic suggestion induced a more substantial impact during the CPT than tDCS. For the Δ-HPT, a we did not observe a significant difference between hypnotic suggestion and tDCS (*F* = 3.14; *P* = 0.08), nor was an order effect observed for either outcome.

**Table 4:** Mixed ANCOVA model to assess the treatment effect between groups on Δ-value of the secondary outcomes: HPT and HPTo (*n* = 24).

		β	SEM	*df*	*t*	*P*-value	CI 95%
**Dependent variable : Δ-heatpain threshold**						
Intercept	2.901	0.801	33.19	3.62	**0.00**	(1.27 to 4.53)
Order of intervention	–0.30	0.870	43.99	–0.35	0.72	(-2.06 to 1.48)
Intervention tDCS	–1.60	0.906	43.76	–1.77	**0.08**	(-3.45 to 0.21)
Hypnotic suggestion 0^reference^							
Δ-BDNF (post intervention minus pre-intervention)	0.006	0.012	42.63	0.20	0.83	(-0.03 to 0.03)
**Dependent variable : Δ-heatpain tolerance (HPTo)**							
Intercept	1.91	0.590	34.48	3.24	**0.00**	(0.71 to 3.11)
Order of intervention	–0.42	0.617	43.37	–0.67	0.50	(-1.66 to 0.83)
Intervention tDCS	–1.45	0.639	43.68	–2.25	**0.02**	(-2.73 to 0.16)
Hypnotic suggestion 0^reference^							
Δ-BDNF (post intervention minus pre-intervention)	–0.005	0.008	43.98	–0.53	0.59	(-0.02 to -0.01)

*Brain derived neurotrophic factor (BDNF); β [beta value show the estimated effect of the intervention (factor) and covariates]; Standard error for mean (SEM); degrees of freedom (df).*

#### Relationships Between State Anxiety vs. Baseline During the Δ-Value of NPS During the CPT and Between Δ-BDNF and Δ-Value of NPS During the CPM Test

**FIGURE 5:**
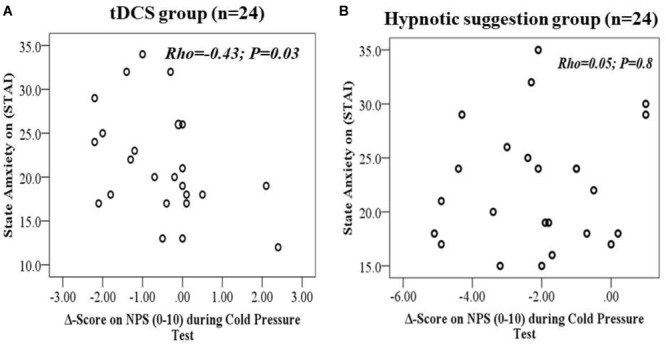
**(A,B)** Scatter plots of State-anxiety with Δ-value of the NPS (0–10) during CPT (i.e., after intervention minus baseline level) according to tDCS **(A)** and hypnotic suggestion **(B)**.

The scatter plots of the raw data for state anxiety and Δ-value of NPS during CPT, according intervention, are shown in Figure 5A,B, respectively. In the tDCS group, state anxiety and the Δ-value of NPS during the CPT showed a negative non-parametric correlation, demonstrating that patients with higher levels of state anxiety that received tDCS treatments showed a lower Δ-value of NPS during the CPT and indicating a larger effect of tDCS in these patients. The Spearman’s correlation coefficient between state anxiety and the Δ-value of NPS during the CPT for the tDCS group was 0.43, with a 95% confidence interval (95% CI) of -0.71 to -0.03 (*P* = 0.03). The Spearman’s correlation coefficient between state anxiety and the Δ-value of NPS during the CPT for the hypnotic suggestion group was 0.05, with a 95% CI of -0.33 to 0.44 (*P* = 0.8).

**FIGURE 6:**
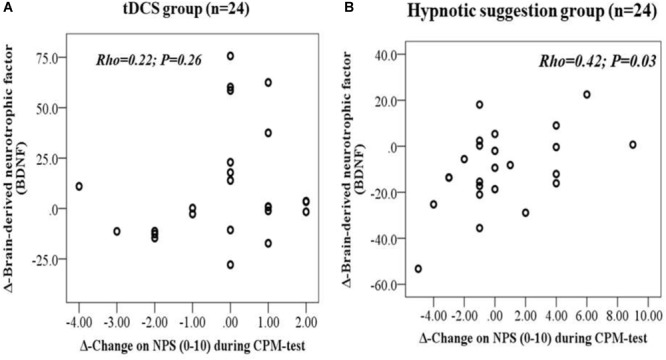
**(A,B)** Scatter plots of Δ-BDNF (post intervention minus pre-intervention) with Δ-Change on NPS (0–10) during CPM-test (i.e., after intervention minus baseline level) according to tDCS **(A)** and hypnotic suggestion **(B)**.

The scatter plots of the raw data for Δ-BDNF and Δ-value of NPS the CPM test, according intervention, are shown in Figure 6A,B, respectively. For the hypnotic suggestion group Δ-BDNF and Δ-value of NPS during the CPM test showed a positive non-parametric correlation, suggesting that patients that received hypnotic suggestions showed a lower Δ-value of NPS during the CPM test. It is important to recognize that higher Δ-values of NPS during the CPM test indicate the reduced potency of the DPMS. The Spearman’s correlation coefficient between Δ-BDNF and Δ-value of NPS during the CPM test was 0.42, with a 95% CI of 0.02–0.70 (*P* = 0.03). The Spearman’s correlation coefficient between Δ-BDNF and Δ-value of NPS during the CPM test in the tDCS group was 0.22, with a 95% CI of -0.20 to 0.57 (*P* = 0.26).

## Discussion

These findings indicate that the effect of hypnotic suggestion on pain perception involves cortical pain processing, whereas tDCS induced either a downregulation of the pain-facilitating pathways or an upregulation of the inhibitory function of the DPMS. In short, these results provide new scientific insights concerning the effects of hypnotic suggestion and tDCS on pain processing, while simultaneously raising important questions regarding the extent to which each intervention can alter pain perception. In addition, an exploratory analysis showed two distinct effects of these two interventions, and increased levels of state anxiety at baseline were correlated with a more substantial tDCS effect on the Δ-value of NPS during the CPT. For hypnotic suggestion, a higher change in the Δ-BDNF value associated with the magnitude of the Δ-value of NPS during the CPM test.

The effects of hypnotic suggestion on CPT and HPTo responses revealed that it was able to decrease pain perception. These results are in line with those reported by randomized controlled studies of clinical populations, which reported that hypnotic suggestion could improve pain conditions and analgesia (Montgomery et al., 2002; Patterson and Jensen, 2003). These results also indicate that hypnotic suggestion might be an effective procedure for alleviating pain perception in experimental models (Vanhaudenhuyse et al., 2009; Brunoni et al., 2016). Due to the complex mechanisms of pain, it is important to investigate the multiple methods through which hypnotic suggestion can influence pain perception, as evaluated by psychophysical pain measures. According to previous studies, the psychophysical measures that are suitable for activating pain pathways, can be measured by valid tests, and are reproducible have a strong probability of being correlated with pain perception. In addition, our results allow the discrimination of effects mediated primarily by cortical mechanisms (i.e., pain threshold and pain tolerance) from those mediated by infra-cortical mechanisms, such as the inhibitory functions of the DPMS, based in the paradigm of the CPM test (da Graca-Tarragó et al., 2015).

In the present study, an exploratory analysis revealed that subjects with increased levels of state anxiety at baseline showed increased responses to anodal tDCS applied to the left DLPFC during the CPT. This result can be explained by the upregulation of reactions to positive emotional stimuli. In accordance with these results, a previous study reported that the anodal stimulation of this region improved the identification of positive emotional expressions (Nitsche et al., 2012). However, other studies found that anodal stimulation of this region may reduce the perceived degree of emotional valence for negative emotional pictures (Peña-Gómez et al., 2011) and expressions of anger in images (De Raedt et al., 2010). In addition, the right DLPFC may be involved in the upregulation of negative emotional outcomes. High-frequency (i.e., excitatory) treatment with repetitive transcranial magnetic stimulation (rTMS) applied to the right DLPFC resulted in impaired attention disengagement from a threat (angry faces) (De Raedt et al., 2010). However, these results remain inconclusive, and further studies are necessary to clarify how the stimulation of both hemispheres affects pain tolerance according to the levels of state anxiety immediately prior to the application of tDCS.

The novelty of these results reveals that hypnotic analgesia induces a dissociative effect, which activates supra-spinal neural networks that reduce pain perception for both heat pain threshold and heat pain tolerance. Conversely, this effect is likely decoupled from the inhibition of the DPMS. When we analyzed the impact of the interventions on Δ-value of NPS during the CPM test within groups (hypnotic suggestion and tDCS) (Table 2), we found small effect sizes for both procedures (hypnotic suggestions ES = 0.22 and tDCS ES = 0.1). However, these effects changed when we adjusted the impact of the interventions by the Δ-BDNF values. The adjusted analysis revealed that hypnotic suggestion improved the inhibitory function of the DPMS. One hypothesis is that hypnotic suggestion reduced the experienced pain level; consequently, the DPMS was less activated by heterotopic painful stimuli. An alternative explanation is that both hypnotic suggestion and painful heterotopic stimuli compete for the same descending inhibitory pathways. In addition, according to the literature, the H-reflex amplitude decreases significantly during hypnosis in highly susceptible subjects, whereas the painful stimulus of the CPM test does not affect this monosynaptic reflex excitability (Santarcangelo, 2014). It is possible that the subjects may have experienced less pain during hypnotic suggestion and resulting in the observed decrement in the Δ-BDNF value. However, we do not have a clear explanation for how hypnotic suggestion affects BDNF secretion or whether the observed effects of hypnotic suggestion on the DPMS are dependent on changes in this neurotrophic factor.

The application of tDCS increased the function of the descending pain inhibitory system when we adjusted for changes in the BDNF levels. This result indicates that the bi-encephalic approach used to apply the tDCS altered neuroplasticity processes and improved the DPMS. Although the mechanisms underlying this effect are not entirely understood, it is plausible that tDCS can activate structures within the brainstem that are involved in the inhibitory function of the DPMS. This result demonstrates that the effects of tDCS on the descending inhibitory pathway may be linked to increased cortical excitability. This hypothesis is supported by evidence from left DLPFC studies, which indicate that the analgesic mechanisms of tDCS involve the activation of top-down downstream circuits to the anterior insula, the hypothalamus, the periaqueductal gray region, the nucleus accumbens and the rostroventral medulla (Wager and Atlas, 2015). Thus, cortical hyperexcitability and increased spinal inhibition could explain the observed changes in BDNF levels. Although these results help improve the understanding of the relationship between tDCS applied to the left DLPFC and serum BDNF and their effects on DPMS function, it is necessary to be judicious when interpreting these results. Because these results were observed in healthy subjects, in an experimental model after, one session of tDCS these findings may not be applicable to other situations. Thus, further studies are necessary.

In the current study, the bi-encephalic tDCS montage was not able to increase the pain threshold for the CPT. Our results are similar to those reported by another study using healthy subjects, where a significant effect on thermal thresholds was not found for tDCS applied to the left DLPFC (Mylius et al., 2012a). However, studies with similar characteristics found considerable increases in the HPT when anodal tDCS was applied to both the M1 (Mylius et al., 2012b; Caumo et al., 2016) and the right DLPFC (Mylius et al., 2012b). Likewise, either low- or high-frequency rTMS applied to the right or left DLPFC has been reported to reduce cold- or heat-induced pain (Graff-Guerrero et al., 2005; Borckardt et al., 2007). Three studies in healthy subjects found that rTMS applied to the right or left DLPFC reduced the sensitivity to thermal pain stimuli (Graff-Guerrero et al., 2005; Nahmias et al., 2009).

Although these results are intriguing, the effect sizes for both interventions were small in this experimental model using healthy subjects. Thus, further clinical studies are required, especially as the clinical administration of these procedures generally involves repeated sessions. In addition, although there are limitations on the translation of an experimental paradigm to the clinical setting, these experiments allow us to characterize the etiological components of pain (e.g., the nature, localization, intensity, frequency and duration of the trigger necessary to evoke pain). Thus, these results add to the body of research regarding the use of hypnotic analgesia in combination with psychophysical pain measures, which are widely used to evaluate the effects of interventions on pain processing. Furthermore, these studies permit the measurement of a dynamic series of multiple neurophysiological mechanisms that modulate pain perception.

There are several concerns related to the design and data interpretation of this study. First, we included only females because the literature has shown that the pain response is increased in females compared to males. The differences between sex on pain perception have been attributed to physiological and psychological variables, including mechanisms of endogenous inhibition, the capability to endure pain, genetic factors, pain expectation and personality traits (Keefe et al., 2000). In addition, females are more prone to respond to negative emotional stimuli (i.e., stress, fear, and anxiety). Thus, sex could be a confounding factor. Second, BDNF levels indicate neuronal activity (Karege et al., 2002), and BDNF is able to cross the blood-brain barrier (BBB); therefore, the peripheral blood level of BDNF is a reliably good indicator of BDNF levels in the brain (Gonçalves et al., 2008). Thus, we assumed that increased serum BDNF levels indicated a diffuse increase in cortical excitability associated with anodal stimulation (Romero Lauro et al., 2014). Third, although it is a crossover design with a small sample, this design can help prevent the overestimation of the benefits of the intervention being tested (Mills et al., 2009). A potential advantage of this design is that it allows the subject to be the control (Jones, 2014). Fourth, we did not observe a “carryover” effect, which means that the effects found for each phase of the experiment do not reflect the impacts of any residual effects of therapy provided during previous phases of the experiment (Freeman, 1989). Fifth, the findings may only apply to subjects with high levels of hypnotic susceptibility. Thus, further research should explore the beneficial aspects of hypnotic suggestion for chronic pain. In addition, these results provide new insights for psychologists, psychotherapists and hypnosis practitioners and suggest that hypnosis may represent an effective treatment for chronic pain, especially when coupled with its cost-effectiveness and minimal side effects. Sixth, although we did not formally measure the potential impact of awareness of the allocation group on the outcomes, a sham intervention that is meaningful for hypnosis is not feasible. Despite these limitations, our findings were evaluated using psychophysical parameters, which are less prone to assessment bias than self-reported measures. Finally, we showed a dissociation between the effects of hypnotic suggestion and DPMS function. These findings provide additional insights into the integration of cortical and distant neural circuits in pain processing. While these results are essential to the understanding of the possible neurobiological mechanisms of hypnotic suggestion on the DPMS compared with tDCS, they do not support therapeutic decision-making in clinical settings.

In conclusion, these results confirm a differential effect between hypnotic suggestion and tDCS on pain measures. They suggest that the impacts of these interventions can be explained by differential effects on contra-regulating mechanisms involved in pain perception, as hypnotic suggestion improved pain tolerance, whereas tDCS increased inhibition in the DPMS. Furthermore, they highlight that Δ-BDNF value influenced the effect of these interventions differently with regards to the inhibitory function of the DPMS; hypnotic suggestion paradoxically decreased the inhibitory function of the DPMS, whereas tDCS increased the inhibitory function of the DPMS. Overall, these findings increase our understanding of the differential effects of these interventions on pain processing, and further studies should be performed that examine their combined effects.

## Data Availability

The raw data supporting the conclusions of this manuscript will be made available by the authors, without undue reservation, to any qualified researcher.

## Ethics Statement

The protocol was approved by the Institutional of Hospital de Clínicas de Porto Alegre. Review Board (IRB Nos. 63863816000005327 and 16-0635) and conducted according to the Declaration of Helsinki. Clinical Trial Registration: www.ClinicalTrials.gov, identifier NCT03744897.

## Author Contributions

GBS conceived the study, participated in its design, in the sequence alignment and coordination, and helped to draft the manuscript. LR, BS, and LdCA participated in the sequence alignment. AS and IT participated in the study design and in the sequence alignment. FF participated in the study design and coordination and helped to draft the manuscript. WC conceived the study, participated in its design, in the sequence alignment and coordination, and helped to draft the manuscript.

## Conflict of Interest Statement

The authors declare that the research was conducted in the absence of any commercial or financial relationships that could be construed as a potential conflict of interest.
