# Supplementary material for: Comparison of Hypnotic Suggestion and Transcranial Direct-Current Stimulation Effects on Pain Perception and the Descending Pain Modulating System: A Crossover Randomized Clinical Trial
Source: Front Neurosci. 2019 Jun 26;13:662. doi: 10.3389/fnins.2019.00662 (PMC6608544; doi:10.3389/fnins.2019.00662)
Supplement: Supplementary file 1 [file Table_1.DOCX]

**Appendix**

**Hypnotic Induction**

**HYPNOTIC INDUCTION**

Please make yourself comfortable. Close your eyes and let yourself relax. Take a few slow deep breaths, and notice that as you exhale, you can feel yourself becoming more and more relaxed. You can continue to relax, as I speak to you...and each time you exhale, you can feel yourself becoming more and more relaxed...more and more relaxed. But no matter how relaxed you become, you will hear my voice, and you will be able to respond to my suggestions. If you become at all uncomfortable, you can readjust your body and make yourself comfortable again, and that won’t get in the way of your experience of hypnosis. If you need to speak to me, you will be able to do so easily, without disrupting your hypnotic experience.

Right now, you might want to relax even more, and as you relax, you may feel a slight tingly feeling and your fingers...or in your toes...and if you do, it can comfort you, because you will know that is a feeling of relaxation that some people have as they begin to experience hypnosis. Let your body relax. Just begin to feel a spreading sense of calm...and peace...letting go all of your cares and concerns... let them drift away, like clouds in the wind...dissipating...breaking up...just relaxing more and more...feeling more and more at peace, more calm.... more comfortable and secure...nothing to bother you... nothing to disturb...more and more deeply relaxed, as your enter I a pleasant comfortable state of hypnosis...becoming so deeply involved in hypnosis that you can have all the experiences you want to have...deep enough to experience whatever you want to experience...but only the experiences you want...just your own experiences.

And you can focus your attention on your toes...your right toe...and your left toe. Let your right toe relax...relax completely...and your left toe...letting your toes relax...more and more...more and more relaxed. And let the relaxation spread from your toes into your feet, and let your feet relax. Let them become more and more relaxed...as you can feel so calm and at ease. And now pay attention to your ankles and to your calves... I wonder if you can begin to let go...let go and relax as you feel perhaps a comfortable sense of warmth in your ankles or in your calves...or perhaps it is a cool and easy feeling...in your right leg or in your left leg. Just let your legs relax...more and more relaxed...more and more completely relaxed.

And the relaxation can spread into your thighs...your thighs can relax more and more...just letting go. And you can let your pelvis relax. Just let it go loose and limp... loose and limp...relaxing more and more. Relax your stomach. Let your stomach become completely relaxed. Notice how it feels, can you let it feel completely relaxed...can you notice this now or a bit later? And let the relaxation spread upward into your chest. Let all the nerves and muscles in your chest relax completely...relaxed...loose and limp...feel the peace spreading as you feel so at ease...so secure...your body and mind so relaxed and at peace. And now your back can relax, and your shoulders. Let yourself feel the relaxation in your back and your shoulders...more and more relaxed...loose and limp...completely relaxed.

Let the relaxation spread through your arms, down into your hands and your fingers. Relax more and more...focus on the feelings in your arms and hands. Do your fingers feel heavier than light, or more light than heavier? Focuses in your right upper arm...right lower arm...your right hand... and fingers...relaxing completely, so relaxed...completely relaxed. And now your left arm...relaxing completely, so relaxed...completely relaxed. I wonder if you can go even deeper now. Deeper and deeper...just as you wish...just as comfortable and as deep as you would like to go.

You might like to imagine being somewhere peaceful and relaxing. I like to imagine lying on a quiet beach on a warm sunny day, with a beautiful blue sky and just a few billowy clouds floating by...I can imagine a feeling of a soft gentle breeze...smelling the salt sea air... but you can imagine being anywhere you like. It might be someplace you’ve been...or someplace you’d like to be. Or just a place in your imagination. It doesn’t matter...all that matters is your comfort...your peace. Wherever it is, it is so peaceful and calm...someplace where you can just be you...where you can feel completely at ease and content. And you can imagine yourself actually being there...seeing, in your mind’s eye, the things that you would see if you were actually there now...feeling the things you would feel...hearing the sounds that you would hear...smelling the smells.

And while you are in your perfect place, I am going to count from one to ten. And with each counted you can drift more and more deeply into hypnosis...more and more...able to experience whatever you want to experience. One...drift, drift and deeper...two...more and more centered, and balanced...three...four...deeper and deeper...five ...six...seven...even deeper than before...so deep that you can experience whatever you wish to experience...eight...nine...ten...very deep now...very deep...completely at one with yourself...completely engrossed.”

From Kirsch, I., Lynn, S.J., & Rhue, J.W. (1993). Introducion to clinical hypnosis. In J.W.Rhue, S.J. Lynn, & Kirsch (Eds), *Handbook of clinical hypnosis* (pp.12-14).Washington, D. C.: American Psychological Association.

*“And from now on, you will no longer feel any more pain ... you will not feel any kind of pain after waking up. Your mind will be able to control the sensations of your whole body ... Your mind controls the sensations of your body ... after you wake up, you will no longer feel any kind of pain ... From now on... and after you wake up you will no longer feel any kind of pain ... I will count from one to ten and you will feel no pain anymore... your mind will control all the sensations of your body and after waking up you will not feel pain.”*

1: “*...you're feeling even more relaxed and comfortable...*”

2: “*...you will get even more relaxed and feeling good...*”

3: “*...feeling better and better, you will not feel pain after you're awake...*”

4: “*...even more relaxed...after waking up,* *a heat stimulus will be placed on your forearm...and your hand in the ice...and you will not feel any kind of pain...*”

5: “*...will be relaxed and painless...*”

6: “*...your mind will control all the sensations of your body...will control it and you will no longer feel pain after you wake...*”

7: “*...you relax even more...feeling very well...you relax and have good sensations...*”

8: “...*very deep, you will not be able to feel any kind of pain in the periphery of your body when you are awake...*”

9: “*...you will not feel pain...your brain controls all the sensations of the periphery of your body...you will not feel any kind of pain...*”

10: “*...your brain now controls all your sensations of your body...and you will not feel pain after you wake up...feel even more relaxed and comfortable...when you wake up you will no longer feel any kind of pain.*”

“*Now you are very relaxed and feeling comfortable, but very soon you will wake up. I will count from one to ten and to each number you will wake up even more...I will count from one to ten, and you will wake up, feeling good...and will no longer feel any kind of pain...I will count from one to ten and you will wake up, and you will not be able to feel any kind of pain in your body...one...you are gradually coming back and feeling your own body...two...you are gradually coming back...and you can remember that you will no longer feel any kind of pain...three...you can slowly feel your body and the energy increasing...four...you are waking up and feeling your body on the chair...five...halfway through...after waking up you will no longer feel any kind of pain...six...feeling good and slowly waking up...seven...slowly feeling the environment and the movements of your body...eight...you are almost awake...when I get to ten you will wake up feeling good and your brain will not allow you to feel any kind of pain...nine...you are waking up and being aware of your surroundings...waking up more and more...ten...now you wake up feeling good...open your eyes.”*

Based on the hypnotic analgesia approach from Jensen, M. P. (2011). *Hypnosis for chronic pain management: Therapist guide*. Oxford University Press.
